# Network analysis of frailty, sleep quality, and life satisfaction in older patients: a cross-sectional study stratified by chronic disease burden

**DOI:** 10.1186/s12877-026-07958-x

**Published:** 2026-08-03

**Authors:** Zhiqiang Wang, Zhanhua Wei, Xiaoya Li, Mengqiu Hu, Kaiqin Deng, Tong Cheng, Limeng Zhao, Xu Han, Zhiru Liang, Jing Wang, Shuping Zhao

**Affiliations:** 1 Health Science Center, Yangtze University, No. 1 Xueyuan Road, Jingzhou, Hubei 434023 P.R. China; 2 Shanghai Sixth People’s Hospital, Shanghai Jiao Tong University School of Medicine, No. 600 Yishan Road, Xuhui District, Shanghai, 200233 P.R. China; 3 Huanggang Central Hospital, No. 6 Qi’an Avenue, Huangzhou District, Huanggang, Hubei 438000 P.R. China; 4 Outpatient Service Center, Jingzhou First People’s Hospital, No. 8 Hangkong Road, Shashi District, Jingzhou, Hubei 434000 P.R. China; 5 Department of Geriatric Medicine, Xilingol League Central Hospital, Inner Mongolia Autonomous Region, No. 9 Nadamu Street, Xilinhot, 026000 P.R. China; 6 Department of Hepatobiliary Surgery, Xilingol League Central Hospital, Inner Mongolia Autonomous Region, No. 9 Nadamu Street, Xilinhot, 026000 P.R. China; 7 Department of Anesthesiology, Xilingol League Central Hospital, Inner Mongolia Autonomous Region, No. 9 Nadamu Street, Xilinhot, 026000 P.R. China

**Keywords:** Older patients, Multimorbidity, Frailty, Sleep quality, Life satisfaction, Network analysis, Chronic disease burden

## Abstract

**Background:**

Multimorbidity and chronic disease burden are common among older patients and are closely associated with frailty, poor sleep, and reduced subjective well-being. However, total-score-based analyses may obscure item-level interactions across health domains. This study examined the network structure of frailty, sleep quality, and life satisfaction in older patients and explored whether association patterns varied across chronic disease burden levels.

**Methods:**

This cross-sectional study included 1,107 older patients. Multimorbidity was defined as the presence of two or more chronic diseases. Frailty, sleep quality, and life satisfaction were assessed using the Fatigue, Resistance, Ambulation, Illnesses, and Loss of Weight (FRAIL) scale, Pittsburgh Sleep Quality Index (PSQI), and Satisfaction With Life Scale (SWLS), respectively. A regularized partial correlation network was estimated using the graphical least absolute shrinkage and selection operator (LASSO) with extended Bayesian information criterion (EBIC) model selection (EBICglasso), with scale items or components as nodes. Strength, expected influence, and bridge centrality were calculated. Stratified analyses were conducted by number of chronic diseases, and bootstrap procedures were used to assess network accuracy and stability.

**Results:**

Multimorbidity was present in 1,078 participants (97.4%), and the median number of chronic diseases was 5 (IQR, 3–6). Network analysis showed that SWLS-related nodes had relatively high strength and bridge strength, indicating relatively high statistical connectivity within the overall network and across communities. Sleep-related nodes, particularly sleep latency, use of sleeping medication, and daytime dysfunction, were prominent in signed centrality indices. Frailty nodes were mainly connected to SWLS nodes through negative edges. The SWLS community showed dense internal connections, whereas frailty and sleep quality were linked to life satisfaction primarily through negative cross-community edges. In descriptive stratified analyses, negative cross-community connections appeared more pronounced in the group with ≥ 5 chronic diseases. Because the cutoff was data-driven and no formal network comparison test was performed, the apparent between-group differences should be interpreted as descriptive and exploratory rather than statistically confirmed. Network stability analyses indicated acceptable stability.

**Conclusions:**

Among older patients, life satisfaction-related nodes showed relatively high overall connectivity, while several sleep-related nodes showed notable statistical associations within the estimated network. Given the high prevalence of multimorbidity in this sample, multidisciplinary assessment and health management should consider the interrelations among sleep problems, frailty, and life satisfaction in the context of chronic disease burden. Network analysis may provide exploratory evidence on cross-domain associations in complex health problems among older patients, but apparent differences across chronic disease burden groups require confirmation using formal network comparison tests and longitudinal designs.

**Supplementary Information:**

The online version contains supplementary material available at https://doi.org/10.1186/s12877-026-07958-x.

## Introduction

Against the backdrop of population aging, multimorbidity has become a persistent clinical issue in geriatric care and multidisciplinary clinical practice [1]. The World Health Organization emphasizes that healthy aging is not merely about prolonging life, but about maintaining intrinsic capacity, functional status, and individualized life goals in older adults [2]. As chronic disease burden increases, older adults with multimorbidity often face complex health needs and care challenges. Accordingly, the National Institute for Health and Care Excellence recommends that multimorbidity management address interactions among diseases and treatments, treatment burden, and patient-centered outcomes such as quality of life [3]. Within this framework, frailty is a key dimension for risk stratification and comprehensive assessment. Frailty reflects reduced physiological reserve and increased vulnerability to stressors and is closely associated with adverse outcomes, including falls, disability, hospitalization, and mortality [4]. Multimorbidity and frailty are interdependent. The accumulation of chronic diseases may increase frailty risk through inflammation, impaired nutrition, reduced activity, medication burden, and functional decline, whereas frailty may weaken disease self-management and worsen health outcomes [5, 6]. Thus, health status in older patients with multimorbidity is usually multidimensional.

Sleep quality and life satisfaction are important dimensions for understanding frailty in older patients with multimorbidity. Sleep disturbances are common in older adults and are intertwined with chronic diseases, pain, psychological symptoms, medication use, and functional limitations [7]. In a systematic review and meta-analysis, Pourmotabbed et al. [8] showed that sleep problems were associated with frailty risk in older adults. Further evidence suggests that both short and long sleep duration are linked to higher incidence or prevalence of frailty [9]. Studies in Chinese older adults also support the potential mediating roles of sleep quality, depressive symptoms, and anxiety symptoms between frailty and multimorbidity [10, 11]. Life satisfaction, as a cognitive component of subjective well-being, reflects individuals’ evaluation of their overall life, living conditions, and past achievements [12]. In older adults, life satisfaction is associated with sleep quality, chronic disease burden, functional status, social support, and mental health [13–15]. A study of community-dwelling and institutionalized older adults showed that sleep quality and frailty explained differences in quality of life, suggesting that physiological vulnerability and sleep problems may jointly affect subjective health outcomes [16]. However, most existing studies have used regression analysis, mediation models, or total-score-based correlations, with limited attention to item-level associations among Fatigue, Resistance, Ambulation, Illnesses, and Loss of Weight (FRAIL) scale items, Pittsburgh Sleep Quality Index (PSQI) components, and Satisfaction With Life Scale (SWLS) items. Total-score models can estimate overall status but may obscure heterogeneity across specific dimensions. For example, fatigue, difficulty walking, sleep latency, daytime dysfunction, use of sleeping medication, and the SWLS item “obtained important things in life” may play different roles in the overall health system; total scores alone cannot clarify which symptoms or subjective evaluations show relatively strong cross-domain associations.

Network analysis provides a methodological approach for examining complex relations among frailty, sleep quality, and life satisfaction in the context of multimorbidity. This method treats variables as nodes and statistical associations adjusted for other nodes as edges, allowing the description of relative node connectivity and cross-domain association patterns [17–19]. The network estimation and stability framework proposed by Epskamp et al. [17] provides a widely used basis for assessing edge-weight accuracy and centrality stability in cross-sectional psychological and health data. The bridge centrality indices proposed by Jones et al. [18] can describe nodes showing relatively strong cross-community associations across symptom communities or health domains. In recent years, network analysis has been used to explore complex links among frailty, depressive symptoms, sleep quality, and cognition-related problems in older adults [20–22]. However, few studies have integrated frailty, sleep quality, and life satisfaction within the same network framework in older patients with multimorbidity. Therefore, this cross-sectional study applied network analysis to older patients to characterize associations among frailty, sleep quality, and life satisfaction, examine relative node connectivity and cross-domain associations, and descriptively explore network patterns across chronic disease burden levels. Given the high prevalence of multimorbidity among older patients, this study examined the overall network structure and exploratory stratified patterns across chronic disease burden levels, aiming to inform multidisciplinary assessment, symptom management, and future longitudinal and intervention studies.

## Methods

### Study design and participants

This cross-sectional study enrolled older patients treated in the Department of Geriatrics, Jingzhou First People’s Hospital, from February to April 2025. This study was reported in accordance with the Strengthening the Reporting of Observational Studies in Epidemiology (STROBE) checklist for cross-sectional studies. The completed STROBE checklist is provided as supplementary material.

Eligible participants were aged ≥ 60 years and were able to complete the questionnaires independently or with standardized assistance. Patients were excluded if they had severe cognitive, consciousness, psychiatric, hearing, visual, or language impairments that prevented valid questionnaire completion; acute or critical conditions that precluded participation; or missing key study variables or clearly abnormal data after data-quality checks.

### Data collection and measures

#### Demographic and clinical characteristics

A standardized questionnaire was used to collect demographic, lifestyle, and clinical information. Demographic variables included sex (male or female), age (continuous, in years), height (cm), weight (kg), body mass index (BMI, kg/m²), educational level, and primary source of financial support. Lifestyle variables included smoking status and alcohol-drinking status. Clinical variables included the types and total number of chronic diseases. BMI was calculated as weight (kg)/height squared (m²) and categorized according to the Chinese adult BMI classification criteria. Chronic diseases were identified from physician-documented diagnoses in admission records and documented medical histories and coded according to the International Classification of Diseases, 10th Revision (ICD-10). Each chronic disease was counted once for each participant.

#### Multimorbidity and chronic disease burden

Multimorbidity was defined as the presence of ≥ 2 chronic diseases [5]. Multimorbidity status was used to describe chronic disease burden in the study population. To explore patterns across chronic disease burden levels, stratified analyses were performed according to the distribution of the number of chronic diseases. Because the median number of chronic diseases in the clinical data was 5, participants were classified into a lower-burden group (< 5 chronic diseases) and a higher-burden group (≥ 5 chronic diseases). This data-driven cutoff was selected to obtain comparably sized groups for exploratory descriptive analyses and does not represent a validated clinical threshold.

#### Frailty assessment

Frailty was assessed using the Chinese version of the FRAIL scale. The FRAIL scale is a brief screening tool for frailty [23], and its Chinese version has undergone cross-cultural adaptation and reliability and validity testing in Chinese older adults, showing good applicability [24]. The scale includes five items: fatigue, resistance/difficulty climbing stairs, ambulation/walking difficulty, illnesses, and weight loss. Each item is scored as 0 or 1, with a total score ranging from 0 to 5; higher scores indicate greater frailty. The “illnesses” item in the FRAIL scale was scored according to the original scale rules for frailty assessment. In contrast, the number of chronic diseases used for chronic disease burden stratification in this study was derived from clinical records; therefore, the two measures were not identical.

In descriptive analyses, the FRAIL total score was calculated to describe overall frailty. In network analysis, the five FRAIL items were entered as frailty nodes: fatigue (F1), resistance/difficulty climbing stairs (F2), ambulation/walking difficulty (F3), illnesses (F4), and weight loss (F5). This approach was used to identify item-level network associations between frailty, sleep quality, and life satisfaction.

#### Sleep quality assessment

Sleep quality was assessed using the Chinese version of the PSQI. The PSQI is widely used in clinical and research settings to assess sleep quality. The original scale contains 19 self-rated items that form seven component scores: subjective sleep quality, sleep latency, sleep duration, habitual sleep efficiency, sleep disturbances, use of sleeping medication, and daytime dysfunction. The seven component scores are summed to generate the PSQI total score, with higher scores indicating poorer sleep quality [25]. The Chinese version of the PSQI has shown acceptable reliability and validity in Chinese populations and has been widely used in sleep-related studies [26].

In descriptive analyses, the PSQI total score was calculated to evaluate overall sleep quality. In network analysis, the seven PSQI components were entered as sleep quality nodes: subjective sleep quality (S1), sleep latency (S2), sleep duration (S3), habitual sleep efficiency (S4), sleep disturbances (S5), use of sleeping medication (S6), and daytime dysfunction (S7). This approach was used to examine the role of specific sleep dimensions in the overall network among older patients.

#### Life satisfaction assessment

Life satisfaction was assessed using the Chinese version of the SWLS. The SWLS measures individuals’ cognitive evaluation of their overall life [12], and its Chinese version has been validated in Chinese populations [27]. The SWLS contains five items, each scored from 1 to 7, with a total score ranging from 5 to 35; higher scores indicate greater life satisfaction.

In descriptive analyses, the SWLS total score was calculated to describe overall life satisfaction. In network analysis, the five SWLS items were entered as life satisfaction nodes: life is close to ideal (L1), life conditions are good (L2), satisfied with life (L3), obtained important things in life (L4), and would change almost nothing if living life over (L5). This approach was used to examine network links between specific aspects of life satisfaction and frailty and sleep quality.

### Data quality control

All data were collected by uniformly trained investigators. Before data collection, the research team trained investigators on the study objectives, survey procedures, scale completion requirements, data verification, and privacy protection. After collection, questionnaires were checked for completeness and logical consistency. Records with obvious entry errors, missing key variables, or failure to meet study requirements were excluded. After data cleaning and quality control, participants who met the inclusion criteria and had complete key variables were included in the statistical analysis.

### Network node specification

Within the network analysis framework, specific items or dimensions of frailty, sleep quality, and life satisfaction were defined as nodes to describe item-level association patterns among health characteristics in older patients. The main network included 17 nodes: five FRAIL nodes, seven PSQI nodes, and five SWLS nodes. Frailty nodes included fatigue (F1), resistance/difficulty climbing stairs (F2), ambulation/walking difficulty (F3), illnesses (F4), and weight loss (F5). Sleep quality nodes included subjective sleep quality (S1), sleep latency (S2), sleep duration (S3), habitual sleep efficiency (S4), sleep disturbances (S5), use of sleeping medication (S6), and daytime dysfunction (S7). Life satisfaction nodes included life is close to ideal (L1), life conditions are good (L2), satisfied with life (L3), obtained important things in life (L4), and would change almost nothing if living life over (L5).

Total scores of the PSQI, FRAIL, and SWLS were not included in the network analysis to avoid information duplication and statistical redundancy between total scores and item or component nodes. Nodes were assigned to three theoretically defined communities: frailty, sleep quality, and life satisfaction. This classification was used for subsequent bridge centrality analysis. Bridge centrality indices were calculated to describe the relative strength of cross-community associations among frailty, sleep quality, and life satisfaction nodes.

In the stratified network analyses, the illnesses node in the FRAIL scale (F4) partially overlapped conceptually with the stratification variable, the number of chronic diseases. To reduce redundant interpretation and limited within-group variability, F4 was excluded from the stratified networks and centrality comparisons, while the remaining 16 nodes were retained. This approach supported more cautious interpretation of the exploratory stratified network results.

### Statistical analysis

R software was used for data management, statistical analysis, and network analysis. The distributions of continuous variables were assessed using histograms and Q–Q plots. Age, height, weight, and BMI were summarized as mean ± standard deviation (SD); the number of chronic diseases and ordinal variables were summarized as median (interquartile range, IQR); and categorical variables were presented as frequency and percentage. Associations between demographic or clinical characteristics and PSQI, FRAIL, and SWLS total scores were analyzed using methods appropriate to variable type. The Mann–Whitney U test was used for binary variables, the Kruskal–Wallis H test for multicategory variables, and Spearman’s correlation for associations between continuous variables and scale scores. All tests were two-sided, and *P* < 0.05 was considered statistically significant.

Because the node variables included binary, ordinal, and Likert-type variables, the correlation matrix was estimated using the cor_auto function. A regularized partial correlation network was then constructed using the graphical least absolute shrinkage and selection operator (LASSO) with extended Bayesian information criterion (EBIC) model selection (EBICglasso), with the EBIC tuning parameter γ set to 0.5.

In the network, edges represented partial correlations between nodes after adjustment for all other nodes. Strength and expected influence were calculated to describe relative node connectivity.

Nodes were further assigned to three communities—frailty, sleep quality, and life satisfaction—and bridge strength and bridge expected influence were calculated to describe cross-community connections. All centrality and bridge centrality indices were standardized and presented as Z scores.

Network accuracy and stability were assessed using bootstrap procedures. Nonparametric bootstrap analysis was used to evaluate edge-weight accuracy and generate 95% confidence intervals (CIs). Case-dropping bootstrap analysis was used to assess the stability of centrality and bridge centrality indices. Networks were re-estimated after gradually removing different proportions of the sample, and correlations between the re-estimated and original centrality indices were calculated to obtain the correlation stability coefficient (CS-coefficient).

To descriptively explore network patterns by chronic disease burden, participants were classified into a lower-burden group (< 5 chronic diseases) and a higher-burden group (≥ 5 chronic diseases). Stratified networks were estimated separately. Because the FRAIL illnesses node (F4) conceptually overlapped with the stratification variable, the number of chronic diseases, and could lead to limited within-group variability, F4 was excluded from the stratified networks and centrality comparisons. Stratified networks were estimated using the same method as the total-sample network and visualized with the same layout and edge-weight scaling. Centrality and bridge centrality indices were calculated for the total sample and both burden groups for descriptive comparison. The cutoff was data-driven rather than clinically validated, and the stratified analysis was exploratory and did not imply causal inference or statistically established between-group differences.

### Ethical considerations

This study was reviewed and approved by the Ethics Committee of Jingzhou First People’s Hospital (approval No. YJ202563) and conducted in accordance with the Declaration of Helsinki. Participation was voluntary, informed consent was obtained before participation, and anonymized data were used only for scientific research.

## Results

### Demographic and clinical characteristics and their associations with sleep quality, frailty, and life satisfaction

A total of 1,276 older patients were initially screened, of whom 1,107 were included in the final analysis after data-quality checks. Among the 1,107 included participants, the mean age was 82.14 ± 7.81 years, and 611 (55.2%) were male. The median number of chronic diseases was 5 (IQR, 3–6). Overall, 1,078 participants (97.4%) had multimorbidity.

PSQI, FRAIL, and SWLS total scores differed significantly by sex (all *P* < 0.001). Female patients had poorer sleep quality, higher frailty levels, and lower life satisfaction. Age was positively correlated with PSQI and FRAIL total scores and negatively correlated with SWLS total score. BMI and BMI category were not significantly associated with any of the three scale scores. PSQI, FRAIL, and SWLS total scores also differed significantly by smoking status, drinking status, education level, and primary source of financial support (all *P* < 0.001).

The number of chronic diseases was positively correlated with PSQI and FRAIL total scores and negatively correlated with SWLS total score. Compared with patients without multimorbidity, those with multimorbidity had higher PSQI and FRAIL total scores and lower SWLS total scores; all differences were statistically significant (all *P* < 0.001). Details are shown in Table 1.

**Table 1:** Demographic and clinical characteristics and their associations with PSQI, FRAIL, and SWLS total scores

Variable	Category	Descriptive statistics	PSQI total score	PSQI test	FRAIL total score	FRAIL test	SWLS total score	SWLS test
Sex	—	—	—	U = 72948.5; *p* < 0.001	—	U = 59158.5; *p* < 0.001	—	U = 48982.0; *p* < 0.001
	Male	611 (55.2%)	6.00 (5.00–9.00)		1.00 (1.00–2.00)		25.00 (21.00–28.00)	
	Female	496 (44.8%)	10.00 (8.00–13.00)		3.00 (2.00–4.00)		16.00 (12.00–21.00)	
Age, years	Mean ± SD	82.14 ± 7.81	r_s_ = 0.231	*p* < 0.001	r_s_ = 0.208	*p* < 0.001	r_s_ = -0.174	*p* < 0.001
Height, cm	Mean ± SD	158.80 ± 7.61	r_s_ = -0.329	*p* < 0.001	r_s_ = -0.417	*p* < 0.001	r_s_ = 0.415	*p* < 0.001
Weight, kg	Mean ± SD	61.55 ± 11.00	r_s_ = -0.205	*p* < 0.001	r_s_ = -0.237	*p* < 0.001	r_s_ = 0.237	*p* < 0.001
BMI, kg/m²	Mean ± SD	24.34 ± 3.59	r_s_ = -0.034	*p* = 0.259	r_s_ = -0.011	*p* = 0.720	r_s_ = 0.009	*p* = 0.766
BMI category	—	—	—	H(3) = 3.20; *p* = 0.362	—	H(3) = 1.82; *p* = 0.610	—	H(3) = 4.41; *p* = 0.220
	Underweight	65 (5.9%)	9.00 (6.00–12.00)		2.00 (1.00–3.00)		20.00 (15.00–23.00)	
	Normal weight	419 (37.9%)	8.00 (5.00–11.00)		2.00 (1.00–3.00)		21.00 (16.00–26.00)	
	Overweight	477 (43.1%)	8.00 (5.00–11.00)		2.00 (1.00–3.00)		21.00 (16.00–26.00)	
	Obese	146 (13.2%)	8.00 (6.00–10.00)		2.00 (1.00–3.00)		20.00 (16.00–26.00)	
Smoking status	—	—	—	H(2) = 98.14; *p* < 0.001	—	H(2) = 132.09; *p* < 0.001	—	H(2) = 159.82; *p* < 0.001
	Never smoker	562 (50.8%)	9.00 (7.00–12.00)		2.00 (1.00–3.00)		18.00 (13.00–23.00)	
	Former smoker	236 (21.3%)	7.00 (5.00–10.00)		2.00 (1.00–2.00)		23.00 (19.00–27.00)	
	Current smoker	309 (27.9%)	7.00 (5.00–9.00)		1.00 (1.00–2.00)		24.00 (20.00–28.00)	
Alcohol drinking status	—	—	—	H(3) = 112.98; *p* < 0.001	—	H(3) = 139.15; *p* < 0.001	—	H(3) = 175.64; *p* < 0.001
	Never drinker	603 (54.5%)	9.00 (7.00–12.00)		2.00 (1.00–3.00)		18.00 (13.00–23.00)	
	Former drinker	229 (20.7%)	7.00 (5.00–10.00)		1.00 (1.00–2.00)		23.00 (20.00–27.00)	
	Current occasional drinker	162 (14.6%)	6.00 (4.00–9.00)		1.00 (1.00–2.00)		24.00 (21.00–27.00)	
	Current regular drinker	113 (10.2%)	7.00 (5.00–9.00)		1.00 (1.00–2.00)		25.00 (20.00–28.00)	
Educational level	—	—	—	H(5) = 255.18; *p* < 0.001	—	H(5) = 391.28; *p* < 0.001	—	H(5) = 399.01; *p* < 0.001
	Illiterate/no formal education	451 (40.7%)	10.00 (8.00–13.00)		3.00 (2.00–4.00)		16.00 (12.00–20.00)	
	Literate but did not complete primary school	143 (12.9%)	8.00 (6.00–10.00)		2.00 (1.00–2.50)		21.00 (18.00–25.00)	
	Primary school	243 (22.0%)	7.00 (5.00–10.00)		2.00 (1.00–2.00)		23.00 (18.00–27.00)	
	Junior high school	114 (10.3%)	6.00 (4.00–8.00)		1.00 (0.00–2.00)		25.00 (22.00–28.00)	
	Senior high school/technical secondary school	98 (8.9%)	5.50 (4.00–7.00)		1.00 (0.00–1.00)		27.00 (24.00–29.00)	
	College degree or above	58 (5.2%)	5.00 (3.00–6.75)		0.00 (0.00–1.00)		28.00 (25.00–31.00)	
Primary source of financial support	—	—	—	H(7) = 144.00; *p* < 0.001	—	H(7) = 178.98; *p* < 0.001	—	H(7) = 203.89; *p* < 0.001
	Financial support from children or relatives	297 (26.8%)	10.00 (7.00–12.00)		2.00 (2.00–4.00)		18.00 (12.00–22.00)	
	Own labor income/farming/rehired employment	93 (8.4%)	6.00 (4.00–9.00)		1.00 (0.00–2.00)		24.00 (21.00–28.00)	
	Retirement pension/employee pension	255 (23.0%)	6.00 (4.00–8.00)		1.00 (0.00–2.00)		25.00 (21.00–29.00)	
	Basic pension under urban and rural resident pension insurance	281 (25.4%)	8.00 (6.00–11.00)		2.00 (1.00–3.00)		20.00 (16.00–25.00)	
	Minimum living allowance/support for extremely poor persons/disability or old-age subsidies and other government assistance	90 (8.1%)	9.00 (8.00–12.00)		2.00 (1.00–3.00)		19.00 (12.00–23.00)	
	Spouse’s income or spouse’s pension	68 (6.1%)	8.00 (5.75–9.25)		2.00 (1.00–3.00)		21.00 (16.00–25.00)	
	Savings/property income/rent/interest	21 (1.9%)	7.00 (4.00–10.00)		1.00 (0.00–2.00)		23.00 (17.00–29.00)	
	Commercial insurance/other sources	2 (0.2%)	8.00 (6.50–9.50)		1.00 (0.50–1.50)		20.50 (20.25–20.75)	
Number of chronic diseases	Median (IQR)	5.00 (3.00–6.00)	r_s_ = 0.522	*p* < 0.001	r_s_ = 0.621	*p* < 0.001	r_s_ = -0.654	*p* < 0.001
Multimorbidity status	—	—	—	U = 5953.5; *p* < 0.001	—	U = 7359.0; *p* < 0.001	—	U = 5932.5; *p* < 0.001
	Non-multimorbidity, < 2 diseases	29 (2.6%)	5.00 (3.00–6.00)		1.00 (0.00–1.00)		27.00 (25.00–31.00)	
	Multimorbidity, ≥ 2 diseases	1078 (97.4%)	8.00 (6.00–11.00)		2.00 (1.00–3.00)		21.00 (15.00–26.00)	

Categorical variables are presented as *n* (%). Age, height, weight, and BMI are presented as mean ± standard deviation (SD); the number of chronic diseases is presented as median (interquartile range, IQR). PSQI, FRAIL, and SWLS total scores are presented as median (IQR) for categorical group comparisons. Associations between binary variables and PSQI, FRAIL, and SWLS total scores were examined using the Mann–Whitney U test. Associations between multicategory variables and PSQI, FRAIL, and SWLS total scores were examined using the Kruskal–Wallis H test. Associations between continuous variables and PSQI, FRAIL, and SWLS total scores were examined using Spearman correlation analysis. Multimorbidity status was defined according to the number of chronic diseases, with ≥ 2 chronic diseases classified as multimorbidity. The Mann–Whitney U value was reported as the smaller of the two group U statistics; therefore, no negative U values were reported

*Abbreviations*: *BMI* body mass index, *PSQI* Pittsburgh Sleep Quality Index, *FRAIL* Fatigue, Resistance, Ambulation, Illnesses, and Loss of Weight scale, *SWLS* Satisfaction With Life Scale

### Descriptive statistics of network nodes

Seventeen nodes were included in the overall network. The valid sample size for each node was 1,107, with a missing rate of 0.0%. Among the frailty nodes, fatigue (F1) and resistance/difficulty climbing stairs (F2) had relatively high prevalence, with 52.3% and 51.3% of patients scoring 1, respectively. Ambulation/walking difficulty (F3) was reported by 47.5% of patients. In contrast, illnesses (F4) and weight loss (F5) had lower prevalence, with 22.6% and 20.3% of patients scoring 1, respectively.

Among the sleep quality nodes, sleep disturbances (S5) had the highest median score of 3.00 (IQR, 2.00–3.00); 68.8% of patients scored 3 on this node, indicating that sleep disturbances were the most prominent sleep problem in this sample. Sleep latency (S2), subjective sleep quality (S1), and daytime dysfunction (S7) had median scores of 1.00 (IQR, 1.00–2.00), 1.00 (IQR, 1.00–2.00), and 1.00 (IQR, 0.00–2.00), respectively. Use of sleeping medication (S6) had the lowest median score of 0.00 (IQR, 0.00–1.00), and 73.6% of patients reported no use of sleeping medication.

SWLS node scores ranged from 1 to 7. The median score was 4.00 for all five SWLS nodes. Satisfied with life (L3) had an IQR of 3.00–6.00, whereas the remaining SWLS nodes had IQRs of 3.00–5.00. Overall, life satisfaction node scores were concentrated around moderate levels. Details are shown in Table 2.

**Table 2:** Descriptive statistics of network nodes

Node code	Domain	Node name	Variable name	Range	Median (IQR)	Score distribution
F1	Frailty	Fatigue	Fatigue score	0.00–1.00	1.00 (0.00–1.00)	Score 0: 528 (47.7%); Score 1: 579 (52.3%)
F2	Frailty	Resistance/difficulty climbing stairs	Resistance/difficulty climbing stairs score	0.00–1.00	1.00 (0.00–1.00)	Score 0: 539 (48.7%); Score 1: 568 (51.3%)
F3	Frailty	Ambulation/walking difficulty	Ambulation/walking difficulty score	0.00–1.00	0.00 (0.00–1.00)	Score 0: 581 (52.5%); Score 1: 526 (47.5%)
F4	Frailty	Illnesses	Illnesses score	0.00–1.00	0.00 (0.00–0.00)	Score 0: 857 (77.4%); Score 1: 250 (22.6%)
F5	Frailty	Weight loss	Weight loss score	0.00–1.00	0.00 (0.00–0.00)	Score 0: 882 (79.7%); Score 1: 225 (20.3%)
S1	Sleep quality	Subjective sleep quality	C1 subjective sleep quality	0.00–3.00	1.00 (1.00–2.00)	Score 0: 225 (20.3%); Score 1: 479 (43.3%); Score 2: 307 (27.7%); Score 3: 96 (8.7%)
S2	Sleep quality	Sleep latency	C2 sleep latency	0.00–3.00	1.00 (1.00–2.00)	Score 0: 253 (22.9%); Score 1: 390 (35.2%); Score 2: 334 (30.2%); Score 3: 130 (11.7%)
S3	Sleep quality	Sleep duration	C3 sleep duration	0.00–3.00	1.00 (0.00–2.00)	Score 0: 392 (35.4%); Score 1: 415 (37.5%); Score 2: 232 (21.0%); Score 3: 68 (6.1%)
S4	Sleep quality	Habitual sleep efficiency	C4 habitual sleep efficiency	0.00–3.00	1.00 (0.00–1.00)	Score 0: 465 (42.0%); Score 1: 369 (33.3%); Score 2: 199 (18.0%); Score 3: 74 (6.7%)
S5	Sleep quality	Sleep disturbances	C5 sleep disturbances	0.00–3.00	3.00 (2.00–3.00)	Score 0: 125 (11.3%); Score 1: 115 (10.4%); Score 2: 105 (9.5%); Score 3: 762 (68.8%)
S6	Sleep quality	Use of sleeping medication	C6 use of sleeping medication	0.00–3.00	0.00 (0.00–1.00)	Score 0: 815 (73.6%); Score 1: 227 (20.5%); Score 2: 59 (5.3%); Score 3: 6 (0.5%)
S7	Sleep quality	Daytime dysfunction	C7 daytime dysfunction	0.00–3.00	1.00 (0.00–2.00)	Score 0: 355 (32.1%); Score 1: 384 (34.7%); Score 2: 279 (25.2%); Score 3: 89 (8.0%)
L1	Life satisfaction	Life is close to ideal	SWLS1 score	1.00–7.00	4.00 (3.00–5.00)	Score 1: 78 (7.0%); Score 2: 145 (13.1%); Score 3: 191 (17.3%); Score 4: 242 (21.9%); Score 5: 224 (20.2%); Score 6: 152 (13.7%); Score 7: 75 (6.8%)
L2	Life satisfaction	Life conditions are good	SWLS2 score	1.00–7.00	4.00 (3.00–5.00)	Score 1: 55 (5.0%); Score 2: 99 (8.9%); Score 3: 213 (19.2%); Score 4: 249 (22.5%); Score 5: 239 (21.6%); Score 6: 164 (14.8%); Score 7: 88 (7.9%)
L3	Life satisfaction	Satisfied with life	SWLS3 score	1.00–7.00	4.00 (3.00–6.00)	Score 1: 38 (3.4%); Score 2: 106 (9.6%); Score 3: 168 (15.2%); Score 4: 244 (22.0%); Score 5: 259 (23.4%); Score 6: 185 (16.7%); Score 7: 107 (9.7%)
L4	Life satisfaction	Obtained important things in life	SWLS4 score	1.00–7.00	4.00 (3.00–5.00)	Score 1: 69 (6.2%); Score 2: 122 (11.0%); Score 3: 191 (17.3%); Score 4: 259 (23.4%); Score 5: 240 (21.7%); Score 6: 141 (12.7%); Score 7: 85 (7.7%)
L5	Life satisfaction	Would change almost nothing if living life over	SWLS5 score	1.00–7.00	4.00 (3.00–5.00)	Score 1: 99 (8.9%); Score 2: 139 (12.6%); Score 3: 219 (19.8%); Score 4: 279 (25.2%); Score 5: 184 (16.6%); Score 6: 131 (11.8%); Score 7: 56 (5.1%)

F1–F5 are item nodes of the FRAIL scale; S1–S7 are the seven component nodes of the PSQI; C1–C7 indicate the corresponding PSQI component scores; L1–L5 are item nodes of the SWLS. Node scores are presented as median (interquartile range, IQR). The score distribution column presents the frequency and percentage of each score level for each node

*Abbreviations*: *FRAIL* Fatigue, Resistance, Ambulation, Illnesses, and Loss of Weight scale, *PSQI* Pittsburgh Sleep Quality Index, *SWLS* Satisfaction With Life Scale, *IQR* interquartile range

### Node centrality and bridge centrality indices

Node centrality and bridge centrality indices were calculated based on the EBICglasso regularized partial correlation network. Life satisfaction nodes showed relatively high connectivity in the overall network. Life is close to ideal (L1) had the highest strength (1.347), followed by obtained important things in life (L4; strength = 1.317), satisfied with life (L3; strength = 1.312), life conditions are good (L2; strength = 1.294), and would change almost nothing if living life over (L5; strength = 1.147). These findings indicate that SWLS-related nodes had relatively high statistical connectivity within the estimated network.

For expected influence, sleep latency (S2) ranked highest (0.326), followed by use of sleeping medication (S6; 0.248), daytime dysfunction (S7; 0.201), habitual sleep efficiency (S4; 0.117), and subjective sleep quality (S1; 0.113). In contrast, illnesses (F4) had the lowest expected influence (-0.579), indicating that sleep quality nodes were prominent in terms of signed edge weights.

Bridge centrality analysis showed that obtained important things in life (L4) had the highest bridge strength (0.856), followed by life conditions are good (L2; 0.855), satisfied with life (L3; 0.830), life is close to ideal (L1; 0.806), and illnesses (F4; 0.688). These nodes showed stronger cross-community connections with nodes from other health domains. The three highest-ranking nodes for bridge expected influence were use of sleeping medication (S6; -0.142), daytime dysfunction (S7; -0.167), and sleep latency (S2; -0.185), suggesting that these sleep-related nodes had distinct signed cross-community associations. Details are shown in Table 3.

**Table 3:** Node centrality and bridge centrality indices

Node code	Domain	Node name	Variable name	Strength	Standardized Strength Z value	Strength rank	Expected influence	Standardized Expected influence Z value	Expected influence rank	Bridge strength	Standardized Bridge strength Z value	Bridge strength rank	Bridge expected influence	Standardized Bridge expected influence Z value	Bridge expected influence rank
F1	Frailty	Fatigue	Fatigue score	0.471	-0.955	16	-0.347	-0.748	13	0.428	-0.241	9	-0.39	0.244	9
F2	Frailty	Resistance/difficulty climbing stairs	Resistance/difficulty climbing stairs score	0.548	-0.728	13	-0.329	-0.684	12	0.455	-0.128	7	-0.423	0.117	11
F3	Frailty	Ambulation/walking difficulty	Ambulation/walking difficulty score	0.505	-0.855	14	-0.235	-0.335	9	0.429	-0.234	8	-0.311	0.553	7
F4	Frailty	Illnesses	Illnesses score	0.707	-0.257	7	-0.579	-1.607	17	0.688	0.845	5	-0.599	-0.568	12
F5	Frailty	Weight loss	Weight loss score	0.498	-0.876	15	-0.286	-0.523	11	0.392	-0.391	10	-0.392	0.237	10
S1	Sleep quality	Subjective sleep quality	C1 subjective sleep quality	0.623	-0.507	10	0.113	0.953	5	0.255	-0.965	14	-0.255	0.771	5
S2	Sleep quality	Sleep latency	C2 sleep latency	0.746	-0.141	6	0.326	1.74	1	0.234	-1.049	17	-0.185	1.041	3
S3	Sleep quality	Sleep duration	C3 sleep duration	0.681	-0.335	8	0.085	0.846	6	0.314	-0.716	12	-0.282	0.665	6
S4	Sleep quality	Habitual sleep efficiency	C4 habitual sleep efficiency	0.606	-0.557	12	0.117	0.964	4	0.246	-1.001	15	-0.243	0.816	4
S5	Sleep quality	Sleep disturbances	C5 sleep disturbances	0.438	-1.052	17	-0.208	-0.235	8	0.323	-0.678	11	-0.323	0.505	8
S6	Sleep quality	Use of sleeping medication	C6 use of sleeping medication	0.647	-0.435	9	0.248	1.449	2	0.258	-0.952	13	-0.142	1.211	1
S7	Sleep quality	Daytime dysfunction	C7 daytime dysfunction	0.611	-0.543	11	0.201	1.275	3	0.243	-1.011	16	-0.167	1.114	2
L1	Life satisfaction	Life is close to ideal	SWLS1 score	1.347	1.635	1	-0.266	-0.449	10	0.806	1.34	4	-0.806	-1.375	14
L2	Life satisfaction	Life conditions are good	SWLS2 score	1.294	1.478	4	-0.417	-1.009	16	0.855	1.546	2	-0.855	-1.567	16
L3	Life satisfaction	Satisfied with life	SWLS3 score	1.312	1.534	3	-0.347	-0.748	14	0.83	1.438	3	-0.83	-1.466	15
L4	Life satisfaction	Obtained important things in life	SWLS4 score	1.317	1.548	2	-0.396	-0.929	15	0.856	1.55	1	-0.856	-1.57	17
L5	Life satisfaction	Would change almost nothing if living life over	SWLS5 score	1.147	1.045	5	-0.133	0.04	7	0.64	0.647	6	-0.64	-0.73	13

Strength represents the sum of the absolute edge weights connected to a given node. Expected influence represents the sum of signed edge weights connected to a given node and therefore retains the direction of positive and negative associations. Bridge strength represents the sum of the absolute edge weights linking a given node to nodes from other communities. Bridge expected influence represents the sum of signed edge weights linking a given node to nodes from other communities and reflects the net directional association of cross-community connections. Both centrality and bridge centrality indices are reported as raw values, standardized z scores, and ranks. The network was estimated using the cor_auto correlation matrix and the EBICglasso regularized partial correlation model, with the EBIC tuning parameter γ set to 0.5. F1–F5 are frailty nodes, S1–S7 are sleep quality nodes, and L1–L5 are life satisfaction nod

*Abbreviations*: *FRAIL* Fatigue, Resistance, Ambulation, Illnesses, and Loss of Weight scale, *PSQI* Pittsburgh Sleep Quality Index, *SWLS* Satisfaction With Life Scale, *EBIC* extended Bayesian information criterion, *LASSO*, least absolute shrinkage and selection operator

### Network structure of frailty, sleep quality, and life satisfaction

The network graph showed relatively dense positive connections among life satisfaction nodes. The strongest positive edges were L1–L5, L3–L4, L1–L4, L1–L3, and L1–L2, indicating high coherence among SWLS items. Within the sleep quality community, several relatively strong positive edges were also observed, mainly S2–S6, S2–S3, S1–S7, and S4–S7, suggesting close links among sleep latency, use of sleeping medication, sleep duration, subjective sleep quality, and daytime dysfunction.

**Fig. 1:**
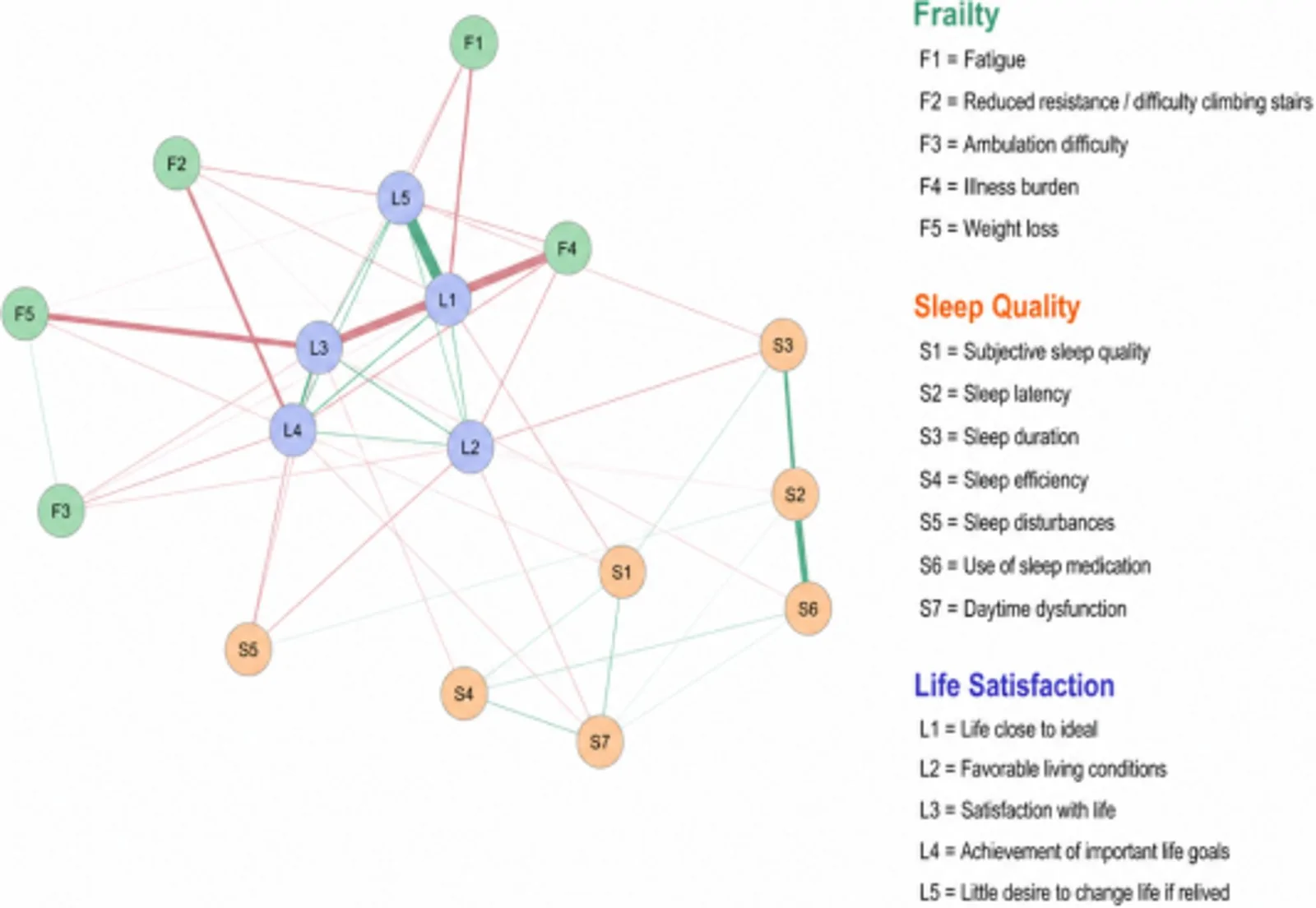
Network structure of frailty, sleep quality, and life satisfaction in older patients. Note: Green nodes indicate frailty, orange nodes indicate sleep quality, and blue–purple nodes indicate life satisfaction. Green edges indicate positive correlations, and red edges indicate negative correlations; thicker edges indicate stronger associations between nodes. The network was estimated using the cor_auto correlation matrix and the EBICglasso regularized partial correlation model, with EBIC γ = 0.5. Node definitions are provided in the figure legend

Cross-community edges were mainly negative, linking frailty and sleep quality nodes to life satisfaction nodes. The stronger negative edges included F4–L3, F5–L3, F4–L1, F2–L4, and F1–L1, indicating negative partial correlations between illnesses, weight loss, resistance/difficulty climbing stairs, fatigue, and life satisfaction nodes. Negative connections also predominated between sleep quality and life satisfaction, particularly S5–L4, S5–L2, S3–L2, and S1–L1. Overall, the life satisfaction community showed the densest internal connections, whereas frailty and sleep quality nodes were linked to life satisfaction mainly through negative cross-community edges. See Fig. 1.

### Visualization of node centrality and bridge centrality

**Fig. 2:**
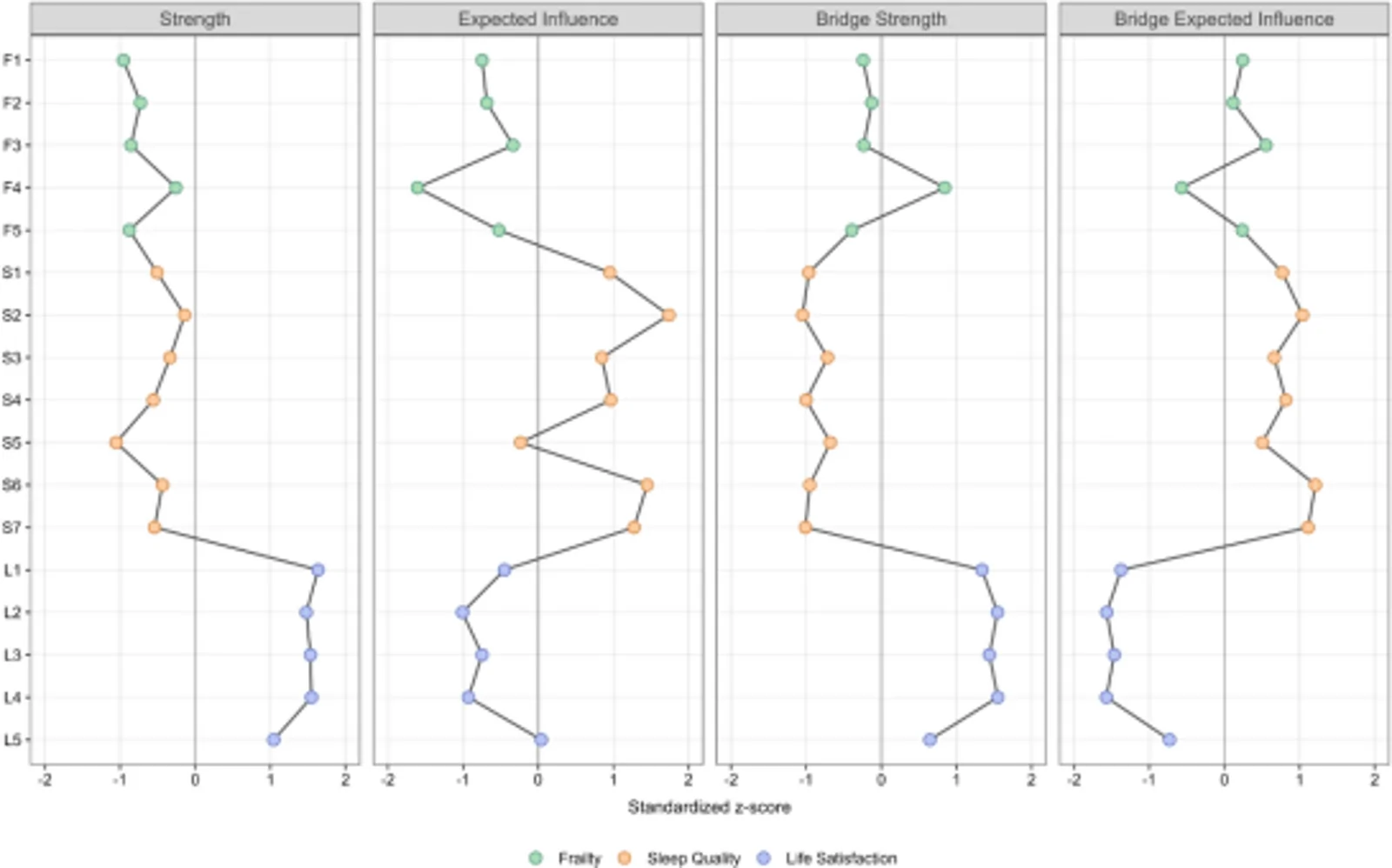
Visualization of node centrality and bridge centrality indices. Note: The figure shows standardized centrality and bridge centrality indices for each network node. Strength is the sum of the absolute edge weights connected to a node. Expected influence is the sum of the signed edge weights connected to a node and reflects the overall influence of both positive and negative edges. Bridge strength is the sum of the absolute edge weights linking a node to nodes in other communities. Bridge expected influence is the sum of the signed edge weights linking a node to nodes in other communities and reflects the net directional association across communities; when the network contains many negative edges, it should be interpreted together with bridge strength. F1–F5 represent frailty nodes, S1–S7 represent sleep quality nodes, and L1–L5 represent life satisfaction nodes. Green, orange, and blue–purple nodes indicate the frailty, sleep quality, and life satisfaction communities, respectively. All indices are presented as standardized z scores. The network was estimated using the cor_auto correlation matrix and the EBICglasso regularized partial correlation model, with EBIC γ = 0.5

To visualize the relative position of each node in the network, plots of node centrality and bridge centrality indices were generated. Figure 2 shows that life satisfaction nodes had overall higher strength and bridge strength, indicating stronger absolute connections with other nodes in the network, particularly nodes from other health domains. This pattern is consistent with Table 3 and further supports the high connectivity of SWLS-related items in the present network.

For expected influence, several sleep quality nodes, especially sleep latency, use of sleeping medication, and daytime dysfunction, ranked relatively high, indicating stronger net associations with other nodes after accounting for edge direction. For bridge expected influence, most cross-community edges were negative. Therefore, this index mainly reflects the net directional association of a node with other health domains and should not be interpreted simply as the magnitude of bridge connectivity. Taken together, bridge strength and bridge expected influence suggest that life satisfaction nodes were characterized mainly by stronger absolute cross-community connections, whereas sleep quality nodes were relatively more prominent in signed cross-community associations.

Overall, Fig. 2 visually supports the findings in Table 3. Life satisfaction nodes formed a highly connected area of the network, whereas sleep quality nodes, particularly sleep latency, use of sleeping medication, and daytime dysfunction, should be regarded as dimensions warranting further clinical assessment and investigation rather than as established intervention targets.

### Accuracy of network edge weights

The main edge-weight estimates showed acceptable precision. Connections within the life satisfaction community were estimated with relatively narrow confidence intervals, particularly the positive edges L1–L5, L3–L4, L1–L4, L1–L3, and L1–L2. Within the sleep quality community, the positive edges S2–S6, S2–S3, S1–S7, and S4–S7 were also estimated with acceptable precision. For cross-community connections, the negative edges F4–L3, F5–L3, F4–L1, F2–L4, and F1–L1 had relatively narrow CIs, indicating comparatively precise estimates of negative associations between frailty-related nodes and life satisfaction nodes. Some weaker edges had wider CIs or crossed 0 and should therefore be interpreted with caution. See Figure S1 and Figure S2 [See Supplementary Materials].

### Stability analysis of network centrality and bridge centrality

**Fig. 3:**
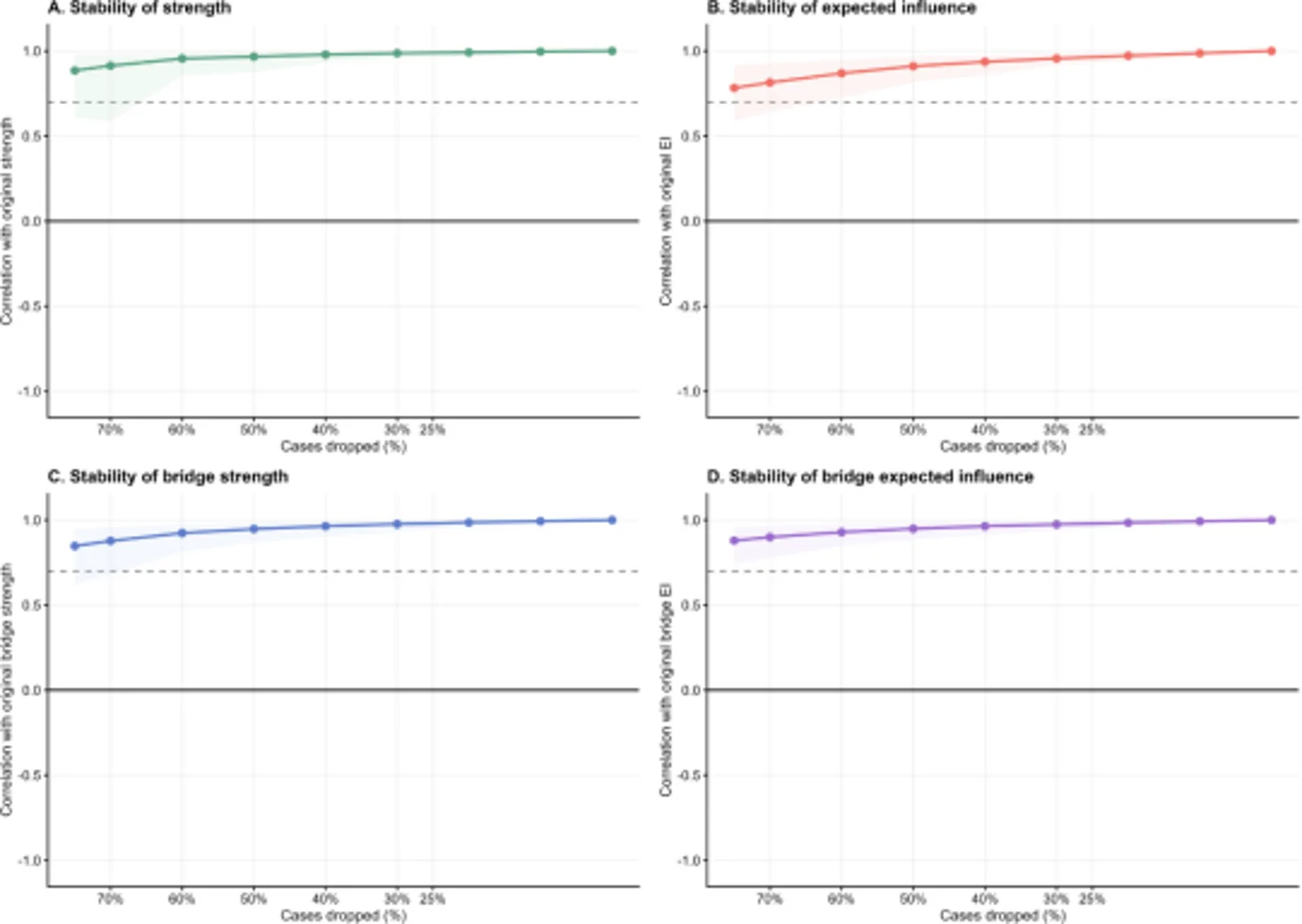
Stability analysis of network centrality and bridge centrality indices. Note: The figure shows the results of case-dropping bootstrap stability analysis. The x-axis indicates the proportion of dropped cases, and the y-axis indicates the correlation between centrality indices from the re-estimated networks and those from the original network. Solid lines indicate mean correlations, shaded areas indicate 95% confidence intervals (CIs), and dashed lines indicate the correlation threshold of 0.70 used in the case-dropping bootstrap analysis. Panels **A**, **B**, **C**, and **D** represent strength, expected influence, bridge strength, and bridge expected influence, respectively. The correlation stability coefficients (CS-coefficients) were 0.75, 0.60, 0.70, and 0.75, respectively. Higher CS-coefficients indicate greater stability of centrality indices under sample perturbation. Abbreviations: CI, confidence interval; CS-coefficient, correlation stability coefficient

As the proportion of dropped cases increased, the correlations between the re-estimated and original indices for strength, expected influence, bridge strength, and bridge expected influence remained generally high. The CS-coefficients were 0.75, 0.60, 0.70, and 0.75, respectively, all exceeding the recommended CS-coefficient threshold of 0.50. These results indicate acceptable stability of both centrality and bridge centrality indices in the network. Even after a relatively large proportion of cases was removed, the re-estimated indices remained strongly correlated with the original network results, supporting cautious interpretation of relative node connectivity and cross-community association patterns. See Fig. 3.

### Stratified networks and centrality comparisons by chronic disease burden

The lower-burden and higher-burden groups included 518 and 589 participants, respectively. In both groups, life satisfaction nodes showed strong positive within-community connections, indicating high consistency among SWLS items across chronic disease burden levels. In descriptive visual comparisons, negative cross-community connections between frailty or sleep quality nodes and life satisfaction nodes appeared more pronounced in the higher-burden group. Because the cutoff was data-driven and no formal network comparison test was performed, these apparent between-group differences should be interpreted as descriptive and exploratory rather than as statistically confirmed differences in network structure.

**Fig. 4:**
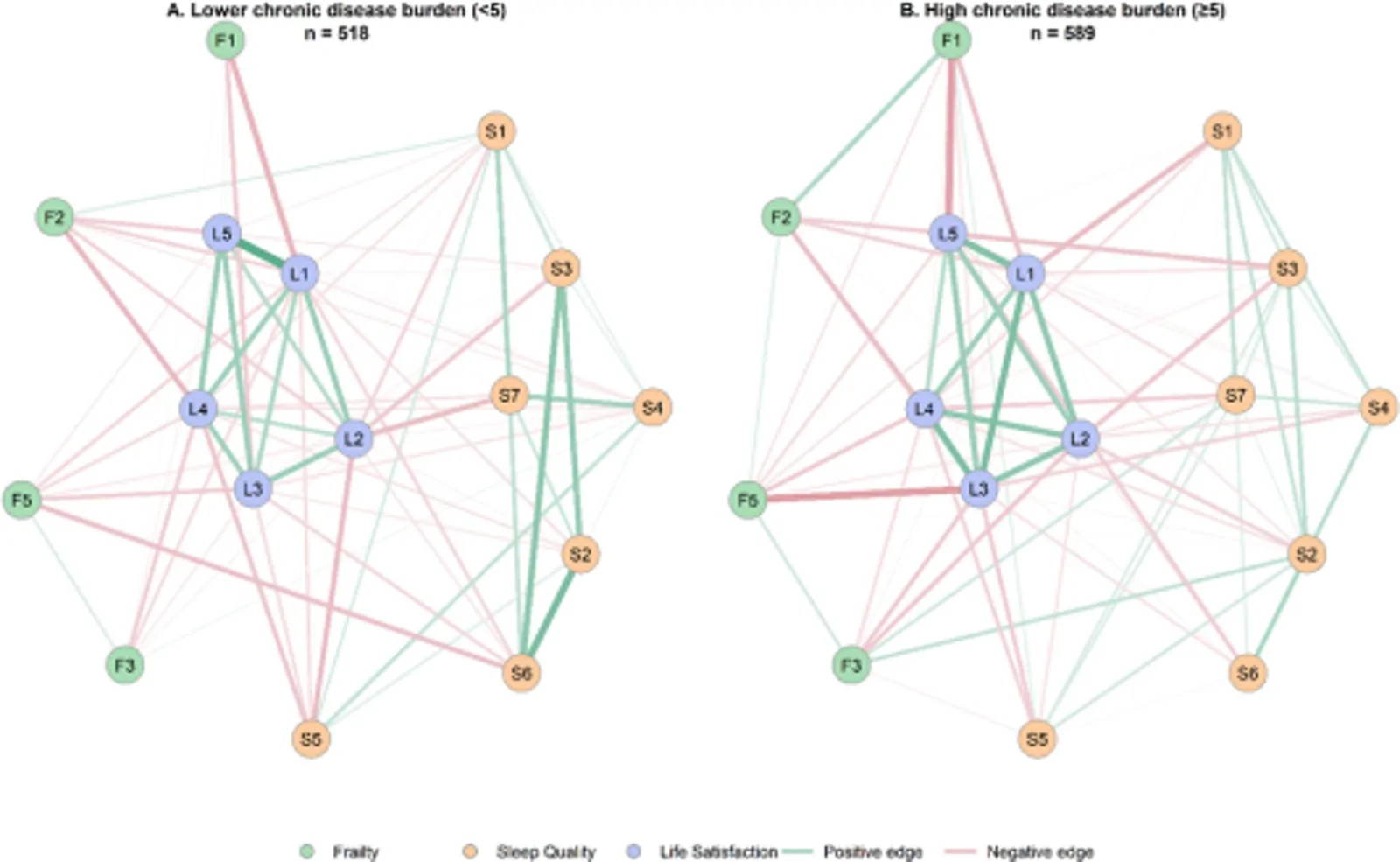
Stratified networks of frailty, sleep quality, and life satisfaction in older patients by chronic disease burden. Note: Fig. 4 shows the stratified network structures for the lower-burden group (< 5 chronic diseases) and the higher-burden group (≥ 5 chronic diseases). Panel **A** represents the lower-burden group, and Panel **B** represents the higher-burden group. Green nodes indicate frailty, orange nodes indicate sleep quality, and blue–purple nodes indicate life satisfaction. Green edges indicate positive correlations, and red edges indicate negative correlations; thicker edges indicate stronger associations between nodes. The two networks were visualized using the same layout and the same edge-weight scaling to facilitate visual comparison. Because the stratification variable was the number of chronic diseases, the illnesses node (F4) was excluded from the stratified network analysis to avoid overlap between the stratification variable and the network nodes and to prevent zero within-group variance. The networks were estimated using the cor_auto correlation matrix and the EBICglasso regularized partial correlation model, with EBIC γ = 0.5

Further descriptive comparison of centrality and bridge centrality across the total sample and stratified networks showed that life satisfaction nodes generally had higher strength and bridge strength in all three networks. In particular, L1–L4-related nodes showed relatively high connectivity in both the total-sample and stratified networks. For expected influence and bridge expected influence, sleep quality nodes, especially S2, S6, and S7, showed notable signed patterns across the networks, suggesting that these sleep-related dimensions warrant further investigation. These findings raise the hypothesis that network associations among frailty, sleep quality, and life satisfaction may vary by chronic disease burden, but this hypothesis requires confirmation using formal network comparison tests and longitudinal designs. See Fig. 4 and Figure S3 [See Supplementary Materials].

## Discussion

### Main findings and network structure

Using network analysis, this study characterized complex associations among frailty, sleep quality, and life satisfaction in older patients. The high prevalence of multimorbidity indicated a substantial chronic disease burden in this sample. Life satisfaction nodes showed relatively high overall connectivity, whereas sleep quality nodes were relatively prominent in signed centrality and bridge indices. Frailty nodes were mainly connected to life satisfaction nodes through negative edges. These findings suggest that subjective life evaluation, sleep status, and physiological functioning are statistically interrelated rather than independent health domains among older patients.

Centrality and bridge centrality indices reflect the relative statistical positioning of nodes within the estimated network. They should not be interpreted as evidence of causality, mechanistic importance, or intervention priority. In this study, life satisfaction nodes showed relatively high strength and bridge strength, while several sleep nodes ranked relatively high in expected influence and bridge expected influence. However, these indices describe relative statistical connectivity within the estimated network and should not be interpreted as evidence that these nodes have greater clinical importance. These findings suggest that different health domains may show distinct association patterns [20, 28]. In the context of multimorbidity, frailty, sleep quality, and life satisfaction may therefore reflect multidimensional associations rather than a pattern explained by a single physiological factor.

### High connectivity of life satisfaction nodes

Life satisfaction nodes (L1–L5) showed relatively high strength and dense internal connections, indicating high statistical connectivity within the estimated network. This finding suggests that SWLS items were closely linked to other nodes and may reflect the integrative nature of life satisfaction in evaluating overall health among older patients. Theoretically, life satisfaction is a cognitive component of subjective well-being and depends on individuals’ integrated appraisal of health, functional status, and social resources [12]. Previous studies have shown stable associations of life satisfaction with functional status, chronic disease burden, and mental health [29–31]. In older adults, life satisfaction is also considered an important component of successful aging and interacts with physical health and social participation [32].

The network showed high coherence among life satisfaction nodes, as reflected by strong edges such as L1–L5 and L3–L4, indicating strong internal coherence within this domain. However, the relatively high centrality of SWLS nodes may partly reflect the internal coherence and conceptual similarity of items belonging to the same psychometric scale. Therefore, higher connectivity of these nodes should not necessarily be interpreted as greater clinical importance. Their broad connections with frailty and sleep nodes may suggest that life satisfaction summarizes multiple dimensions of health status, but this interpretation should remain cautious. This finding is consistent with recent network-based studies showing that subjective health indicators often have high centrality in multidimensional health networks, reflecting their integrative role in the system [33]. One possible explanation is that life satisfaction may reflect an integrated appraisal of health, functional status, emotional well-being, and social resources. However, the cross-sectional design does not allow the direction of these associations to be determined [34].

### Network characteristics of sleep quality nodes

Sleep latency (S2), use of sleeping medication (S6), and daytime dysfunction (S7) ranked relatively high in expected influence and bridge expected influence. These findings indicate that these sleep dimensions warrant attention during comprehensive assessment and further investigation in longitudinal studies. However, bridge expected influence is a signed index and mainly reflects the net directional association of cross-community edge weights; it should not be interpreted as the magnitude of bridge connectivity. When considered together with bridge strength, life satisfaction nodes showed stronger absolute cross-community connections, whereas sleep-related nodes should not be interpreted as established intervention targets.

This pattern is partly supported by previous evidence. Sleep problems are common in older adults and are intertwined with chronic diseases, pain, psychological symptoms, medication use, and functional limitations [7]. Prior studies have shown that poorer sleep quality, difficulty initiating sleep, and daytime dysfunction are associated with frailty risk, functional decline, and lower subjective health evaluations [8, 35, 36]. In this study, sleep latency and daytime dysfunction were relatively prominent in the network, suggesting that sleep problems may reflect not only nighttime sleep status but also daytime functioning and life experience. Use of sleeping medication also showed specific network features and may reflect sleep problem severity, medication burden, and functional risk among older patients [37].

These findings suggest that sleep problems, particularly difficulty initiating sleep, use of sleeping medication, and daytime dysfunction, should be considered together with frailty and life satisfaction in multidisciplinary assessment. Because this study used a cross-sectional design, the network results reflect statistical associations among nodes and cannot establish causal directions among sleep problems, frailty, and life satisfaction. Longitudinal and intervention studies are needed to clarify the role of sleep-related dimensions in comprehensive health management for older patients.

### Negative network associations between frailty and life satisfaction

The network showed negative frailty–life satisfaction connections with comparatively narrow confidence intervals, particularly for fatigue, resistance/difficulty climbing stairs, and illnesses. This finding indicates negative associations between specific frailty manifestations and subjective life evaluation. Previous studies have confirmed significant associations between frailty and poorer quality of life [38, 39]. At the item level, this study further showed that fatigue (F1) and functional limitations (F2 and F3) were connected to multiple dimensions of life satisfaction, suggesting that these symptoms may be relevant to understanding the link between frailty and subjective life evaluation. The illnesses node (F4) showed relatively high bridge strength, indicating cross-community associations between disease burden information and different health domains. This finding is consistent with the cumulative health burden model, in which an increasing number of chronic diseases may be associated with functional status and mental health through multiple pathways [6]. Possible pathways may include reduced social participation, persistent physical discomfort, and fatigue; however, these explanations require further longitudinal investigation [40].

### Descriptive differences in network structure by chronic disease burden

In the descriptive stratified analyses, negative cross-community connections among frailty, sleep quality, and life satisfaction appeared more pronounced in the group with ≥ 5 chronic diseases. Because the cutoff was data-driven and no formal network comparison test was conducted, these apparent differences should be interpreted as descriptive and exploratory rather than as statistically established differences in network structure between chronic disease burden groups. Previous studies have reported cumulative effects of multimorbidity on functional decline, depression, and reduced quality of life [41–43]. The descriptive findings raise the hypothesis that cross-domain associations may vary across chronic disease burden levels. This hypothesis requires validation using formal network comparison tests and longitudinal study designs. From a clinical perspective, these exploratory findings suggest that older patients with a higher chronic disease burden may warrant more integrated assessment of frailty, sleep problems, and life satisfaction, although this implication requires confirmation in longitudinal and intervention studies.

### Implications for multidisciplinary clinical practice and future research

The findings may inform multidisciplinary assessment and hypothesis generation for future studies. First, the relatively high connectivity of life satisfaction nodes suggests that subjective life evaluation may provide complementary information during comprehensive geriatric assessment [12, 29]. Second, sleep assessment should extend beyond a general question about sleep quality to include sleep latency, use of sleeping medication, and daytime dysfunction [7, 8, 37]. Medication use may warrant particular attention from physicians, pharmacists, and nurses because it can reflect both sleep-related needs and treatment burden. Third, frailty-related symptoms, including fatigue and functional limitations, may be considered together with sleep problems and life satisfaction rather than assessed in isolation [4, 38]. These findings support integrated assessment but do not establish intervention priorities.

### Methodological significance and limitations

This study used network analysis to characterize item-level structures among frailty, sleep quality, and life satisfaction, providing an additional perspective for understanding health among older patients in the context of multimorbidity. Compared with total-score-based analyses, this approach can describe item-level association patterns and generate hypotheses regarding cross-domain relationships. However, relative node connectivity should not be interpreted as evidence that a node is a causal mechanism or an intervention priority. Several limitations should be noted. First, the cross-sectional design precludes causal inference, and centrality indices should not be interpreted as evidence of mechanistic importance or intervention priority. Second, the sample was recruited from a single geriatric department and had a high prevalence of multimorbidity, which may limit generalizability to community-dwelling older adults, populations with lower chronic disease burden, and older adults in other healthcare settings. Third, the cutoff of < 5 versus ≥ 5 chronic diseases was based on the sample median rather than a validated clinical threshold, and no formal between-group network comparison test was conducted; therefore, the stratified findings should be interpreted as exploratory. Fourth, the FRAIL illnesses item (F4) partially overlaps conceptually with the number of chronic diseases. Although F4 was excluded from the stratified analyses, residual overlap may still affect interpretation of the overall-sample network. Fifth, the measures were primarily based on self-report scales, which may introduce information bias. Finally, residual confounding may have influenced the estimated network structure. Variables such as age, sex, educational level, medication burden, disease severity, depressive symptoms, functional status, and biological indicators were not explicitly incorporated into the network model. Future studies could integrate longitudinal designs, formal network comparison tests, and additional clinical, functional, psychological, and biological indicators to further validate these exploratory findings.

## Conclusions

Among older patients, life satisfaction nodes showed relatively high connectivity within the estimated network, while sleep latency, use of sleeping medication, and daytime dysfunction showed notable signed associations. Frailty nodes were primarily connected to life satisfaction nodes through negative edges. Descriptive stratified analyses suggested that cross-domain association patterns may vary across chronic disease burden levels. However, because no formal network comparison test was performed, these apparent differences should be interpreted as descriptive and exploratory rather than statistically confirmed, and they require confirmation using formal network comparison tests and longitudinal designs. Multidisciplinary assessment may benefit from considering frailty, sleep problems, and life satisfaction together rather than in isolation.

## Supplementary Information

Supplementary Material 1.

Supplementary Material 2.

## Data Availability

The datasets generated and/or analyzed during the current study are not publicly available because they contain clinical information and participant privacy-related data. Anonymized data may be available from the corresponding author upon reasonable request and subject to approval by the relevant ethics committee and institutional data-management requirements.

## Abbreviations

BMI: body mass index
PSQI: Pittsburgh Sleep Quality Index
FRAIL: Fatigue, Resistance, Ambulation, Illnesses, and Loss of Weight scale
SWLS: Satisfaction With Life Scale
EBIC: extended Bayesian information criterion
LASSO: least absolute shrinkage and selection operator
CI: confidence interval
IQR: interquartile range
ICD-10: International Classification of Diseases, 10th Revision
STROBE: Strengthening the Reporting of Observational Studies in Epidemiology
CS-coefficient: correlation stability coefficient
