# Supplementary material for: Network analysis of frailty, sleep quality, and life satisfaction in older patients: a cross-sectional study stratified by chronic disease burden
Source: BMC Geriatr. 2026 Aug 3;26:1365. doi: 10.1186/s12877-026-07958-x (PMC13721440; doi:10.1186/s12877-026-07958-x)
Supplement: Supplementary file 1 [file 12877_2026_7958_MOESM1_ESM.docx]

Figure S1. Nonparametric bootstrap 95% confidence intervals of nonzero network edge weights

**Note: The figure presents the nonparametric bootstrap 95% confidence intervals (CIs) of nonzero edge weights in the final network. The x-axis represents edge weight. Gray horizontal lines indicate bootstrap 95% CIs; green points indicate positive edges in the original sample; red points indicate negative edges in the original sample; and black points indicate bootstrap means. Labels on the left identify the corresponding node pairs. Edges with narrower CIs indicate comparatively precise estimates, whereas edges with wider CIs should be interpreted cautiously. CIs crossing 0 indicate uncertainty regarding the presence or direction of the corresponding association. The network was estimated using the cor_auto correlation matrix and the EBICglasso regularized partial correlation model, with EBIC γ = 0.5. The nonparametric bootstrap 95% CIs for all possible edge weights are shown in Figure S2.**

**Abbreviations: CI, confidence interval; EBIC, extended Bayesian information criterion.**

Figure S2. Nonparametric bootstrap 95% confidence intervals of all possible edge weights

**Note:** The figure presents the nonparametric bootstrap 95% confidence intervals (CIs) for all possible edge weights in the network. The x-axis represents edge weight. Gray horizontal lines indicate bootstrap 95% CIs, and points indicate edge-weight estimates. Edges with narrower CIs indicate more precise estimates, whereas edges with wider CIs should be interpreted with caution. The network was estimated using the cor_auto correlation matrix and the EBICglasso regularized partial correlation model, with EBIC γ = 0.5.

**Abbreviations:** CI, confidence interval; EBIC, extended Bayesian information criterion.

Figure S3. Comparison of node centrality and bridge centrality across the total sample and chronic disease burden groups

**Note:** Figure S3 compares node centrality and bridge centrality indices across the total sample, the lower-burden group, and the higher-burden group. Panels A–D represent strength, expected influence, bridge strength, and bridge expected influence, respectively. The x-axis shows standardized Z scores within each network, and the y-axis shows network nodes. Black circles represent the total sample, green triangles represent the lower-burden group, and orange squares represent the higher-burden group. Dashed lines indicate a standardized Z score of 0. Strength is the sum of the absolute edge weights connected to a node. Expected influence is the sum of the signed edge weights connected to a node. Bridge strength and bridge expected influence represent the sum of the absolute edge weights and the sum of the signed edge weights, respectively, linking a node to nodes in other communities. Consistent with Figure 4, F4 was excluded from this figure. This figure was used for descriptive comparison of centrality patterns across chronic disease burden levels. Node definitions are the same as those in Figure 1.
