# Supplementary material for: Network analysis of frailty, sleep quality, and life satisfaction in older patients: a cross-sectional study stratified by chronic disease burden
Source: BMC Geriatr. 2026 Aug 3;26:1365. doi: 10.1186/s12877-026-07958-x (PMC13721440; doi:10.1186/s12877-026-07958-x)
Supplement: Supplementary file 2 [file 12877_2026_7958_MOESM2_ESM.docx]

**STROBE Statement—Checklist of items that should be included in reports of cross-sectional studies**

*Manuscript: Network Analysis of Frailty, Sleep Quality, and Life Satisfaction in Older Patients: A Cross-Sectional Study Stratified by Chronic Disease Burden*

| **Section** | **Item No.** | **Recommendation** | **Reported on page(s) / manuscript location** |
| --- | --- | --- | --- |
| Title and abstract | 1(a) | Indicate the study's design with a commonly used term in the title or the abstract. | Reported on p. 1 (title: “A Cross-Sectional Study”) and p. 2 (Abstract, Methods). |
| Title and abstract | 1(b) | Provide in the abstract an informative and balanced summary of what was done and what was found. | Reported on p. 2 (Abstract: Background, Methods, Results, and Conclusions). |
| Introduction | 2 | Explain the scientific background and rationale for the investigation being reported. | Reported on pp. 3–4 (Introduction). |
| Introduction | 3 | State specific objectives, including any prespecified hypotheses. | Reported on p. 4 (final paragraph of the Introduction). No prespecified hypothesis was stated; the stratified analysis was framed as exploratory. |
| Methods | 4 | Present key elements of study design early in the paper. | Reported on p. 4 (Methods, Section 2.1 Study design and participants). |
| Methods | 5 | Describe the setting, locations, and relevant dates, including periods of recruitment, exposure, follow-up, and data collection. | Reported on p. 4 (Methods, Section 2.1): Department of Geriatrics, Jingzhou First People’s Hospital; recruitment from February to April 2025. |
| Methods | 6(a) | Give the eligibility criteria, and the sources and methods of selection of participants. | Reported on p. 4 (Methods, Section 2.1) and p. 6 (Section 2.3 Data quality control). |
| Methods | 7 | Clearly define all outcomes, exposures, predictors, potential confounders, and effect modifiers. Give diagnostic criteria, if applicable. | Reported on pp. 4–8 (Methods, Sections 2.2–2.5). Multimorbidity and chronic disease burden are defined on p. 5; network nodes and stratification procedures are defined on pp. 6–8. |
| Methods | 8* | For each variable of interest, give sources of data and details of methods of assessment (measurement). Describe comparability of assessment methods if there is more than one group. | Reported on pp. 4–6 (Methods, Sections 2.2.1–2.2.5): demographic and clinical data, ICD-10-coded chronic diseases, FRAIL, PSQI, and SWLS. The same assessment methods were used for both burden groups. |
| Methods | 9 | Describe any efforts to address potential sources of bias. | Reported on p. 6 (Section 2.3 Data quality control) and pp. 25–26 (Section 4.7 Methodological significance and limitations). |
| Methods | 10 | Explain how the study size was arrived at. | Not explicitly reported in the current manuscript. The final analytic sample after screening is reported on p. 9. |
| Methods | 11 | Explain how quantitative variables were handled in the analyses. If applicable, describe which groupings were chosen and why. | Reported on p. 5 (Section 2.2.2: <5 vs ≥5 chronic diseases, based on the sample median), pp. 7–8 (Section 2.5), and pp. 10–11 (Table 1). |
| Methods | 12(a) | Describe all statistical methods, including those used to control for confounding. | Reported on pp. 7–8 (Section 2.5 Statistical analysis). Network edges were estimated as partial correlations after adjustment for all other nodes. |
| Methods | 12(b) | Describe any methods used to examine subgroups and interactions. | Reported on p. 5 (Section 2.2.2) and pp. 7–8 (Section 2.5): exploratory stratified networks for the lower-burden and higher-burden groups. |
| Methods | 12(c) | Explain how missing data were addressed. | Reported on p. 6 (Section 2.3 Data quality control). Records with missing key variables were excluded. Node-level missingness is reported as 0.0% on p. 12. |
| Methods | 12(d) | If applicable, describe analytical methods taking account of sampling strategy. | Not applicable. No complex sampling strategy was used. |
| Methods | 12(e) | Describe any sensitivity analyses. | Not applicable / not performed. Bootstrap analyses were used to assess network accuracy and stability rather than as sensitivity analyses (pp. 7–8, 19–20). |

| **Section** | **Item No.** | **Recommendation** | **Reported on page(s) / manuscript location** |
| --- | --- | --- | --- |
| Results | 13(a)* | Report numbers of individuals at each stage of study—e.g., numbers potentially eligible, examined for eligibility, confirmed eligible, included in the study, completing follow-up, and analysed. | Reported on p. 9 (Section 3.1): 1,276 patients were initially screened and 1,107 were included in the final analysis. |
| Results | 13(b)* | Give reasons for non-participation at each stage. | Partially reported. General exclusion criteria and data-quality exclusions are described on pp. 4 and 6, but stage-specific counts for each reason are not itemized. |
| Results | 13(c)* | Consider use of a flow diagram. | Not included in the current manuscript. |
| Results | 14(a)* | Give characteristics of study participants (e.g., demographic, clinical, social) and information on exposures and potential confounders. | Reported on pp. 9–11 (Section 3.1 and Table 1). |
| Results | 14(b)* | Indicate number of participants with missing data for each variable of interest. | Reported in part on p. 12 (Section 3.2): valid sample size for each network node was 1,107 with a missing rate of 0.0%. Data-quality handling is described on p. 6. |
| Results | 15* | Report numbers of outcome events or summary measures. | Reported on pp. 9–14 (Sections 3.1–3.3; Tables 1–3) and pp. 17–22 (network figures and related results). |
| Results | 16(a) | Give unadjusted estimates and, if applicable, confounder-adjusted estimates and their precision (e.g., 95% confidence interval). Make clear which confounders were adjusted for and why they were included. | Reported on pp. 7–8 (Section 2.5 Statistical analysis) and pp. 9–22 (Results). Table 1 presents bivariate associations. Network edges represent regularized partial correlations conditional on the remaining nodes. Edge-weight accuracy is reported on pp. 19–20 and in Supplementary Figures S1–S2. |
| Results | 16(b) | Report category boundaries when continuous variables were categorized. | Reported on p. 5 (Section 2.2.2: <5 vs ≥5 chronic diseases) and pp. 10–11 (Table 1: BMI categories and multimorbidity definition). |
| Results | 16(c) | If relevant, consider translating estimates of relative risk into absolute risk for a meaningful time period. | Not applicable. The study did not estimate relative risks. |
| Results | 17 | Report other analyses done—e.g., analyses of subgroups and interactions, and sensitivity analyses. | Reported on pp. 21–22 (Section 3.8 and Figure 4) and Supplementary Figure S3: exploratory stratified network comparisons. |
| Discussion | 18 | Summarise key results with reference to study objectives. | Reported on p. 23 (Section 4.1 Main findings and network structure) and p. 27 (Conclusions). |
| Discussion | 19 | Discuss limitations of the study, taking into account sources of potential bias or imprecision. Discuss both direction and magnitude of any potential bias. | Reported on pp. 25–26 (Section 4.7 Methodological significance and limitations). |
| Discussion | 20 | Give a cautious overall interpretation of results considering objectives, limitations, multiplicity of analyses, results from similar studies, and other relevant evidence. | Reported on pp. 23–27 (Discussion and Conclusions). Causal and intervention-priority interpretations are explicitly avoided. |
| Discussion | 21 | Discuss the generalisability (external validity) of the study results. | Reported on pp. 25–26 (Section 4.7): single-center geriatric-department sample and high prevalence of multimorbidity may limit generalisability. |
| Other information | 22 | Give the source of funding and the role of the funders for the present study and, if applicable, for the original study on which the present article is based. | Reported on p. 28 (Declarations, Funding): no specific grant was received. |

* Give information separately for exposed and unexposed groups, where applicable. Page numbers refer to the submitted manuscript version used to prepare this checklist. “Not applicable” and “not explicitly reported” entries are stated transparently to avoid overstating the contents of the manuscript.

Note: The STROBE checklist is intended to improve transparent reporting. It does not assess study quality or prescribe a specific study design.
